# Genetic association between smoking and DLCO in idiopathic pulmonary fibrosis patients

**DOI:** 10.1186/s12890-024-02974-2

**Published:** 2024-04-03

**Authors:** Ziheng Yuan, Wanyang Lei, Xiqian Xing, Xiaohua He, Xiaoxian Huang, Li Wei, Yuanyuan Lv, Shuyi Qiu, Ziyu Yuan, Jiyang Wang, Mei Yang

**Affiliations:** 1 Department of Clinical Laboratory, Yunnan Molecular Diagnostic Center, The Second Affiliated Hospital of Kunming Medical University, Kunming, Yunnan China; 2 Department of Respiratory and Critical Care, Affiliated Hospital of Yunnan University, Kunming, China; 3 Department of Clinical Laboratory, The First Affiliated Hospital of Kunming Medical University, Kunming, China; 4 Department of Clinical Laboratory Medicine, Yunnan Cancer Hospital, Yunnan Cancer Center, The Third Affiliated Hospital of Kunming Medical University, 650118 Kunming, China; 5 Department of Cardiovascular Surgery, Affiliated Hospital of Yunnan University, Kunming, China

**Keywords:** Idiopathic pulmonary fibrosis (IPF), Smoking, Diffusing capacity of the lungs for carbon monoxide (DLCO), Mendelian randomization (MR), Causal effect

## Abstract

**Background:**

Observational studies have shown that smoking is related to the diffusing capacity of the lungs for carbon monoxide (DLCO) in individuals with idiopathic pulmonary fibrosis (IPF). Nevertheless, further investigation is needed to determine the causal effect between these two variables. Therefore, we conducted a study to investigate the causal relationship between smoking and DLCO in IPF patients using two-sample Mendelian randomization (MR) analysis.

**Methods:**

Large-scale genome-wide association study (GWAS) datasets from individuals of European descent were analysed. These datasets included published lifetime smoking index (LSI) data for 462,690 participants and DLCO data for 975 IPF patients. The inverse-variance weighting (IVW) method was the main method used in our analysis. Sensitivity analyses were performed by MR‒Egger regression, Cochran’s Q test, the leave-one-out test and the MR-PRESSO global test.

**Results:**

A genetically predicted increase in LSI was associated with a decrease in DLCO in IPF patients [OR_IVW_ = 0.54; 95% CI 0.32–0.93; *P* = 0.02].

**Conclusions:**

Our study suggested that smoking is associated with a decrease in DLCO. Patients diagnosed with IPF should adopt an active and healthy lifestyle, especially by quitting smoking, which may be effective at slowing the progression of IPF.

**Supplementary Information:**

The online version contains supplementary material available at 10.1186/s12890-024-02974-2.

## Introduction

Idiopathic pulmonary fibrosis (IPF) is a progressive interstitial lung disease (ILD) of unknown origin and has a poor prognosis [1]. Epidemiological studies reveal that the global incidence and prevalence of IPF are increasing annually, and the median survival duration following an IPF diagnosis is within the range of 3 to 5 years, with a five-year survival rate of under 30% [2, 3].

Cigarette smoking (CS) is a lifestyle factor that can potentially be modified, which consistently ranks as one of the primary risk factors for IPF. Therefore, CS have garnered significant attention as promising areas of intervention in efforts to prevent the risk of IPF and halt its progression [4]. However, research on the specific role of smoking in driving disease progression in individuals with IPF is still limited. diffusing capacity of the lung for carbon monoxide (DLCO) is an important indicator used to assess disease progression in patients with IPF and reflects the ability of the lungs of IPF patients to transfer gas [5, 6]. Previous observational studies have shown that both current smoking status and increasing pack-years of CS were linked to lower DLCO. This implies that individuals with a history of CS, particularly those who have smoked for an extended period or at a higher intensity, are more susceptible to a decline in DLCO [7–10]. In summary, smoking appears to be a contributing factor to decreased DLCO in patients diagnosed with IPF. However, the determination of smoking as a causal factor of decreased DLCO remains uncertain, given that these studies are primarily observational. These studies are susceptible to confounding bias and reverse causality, which can complicate the interpretation of the relationship between smoking and DLCO in patients with IPF.

Mendelian randomization (MR) is a new approach that addresses these above challenges by using genetic variants as a reliable tool for establishing causal relationships; this approach is less vulnerable to confounding bias than is conventional observational studies [11]. In this context, we conducted an MR study to explore the potential causal link between smoking and a decrease in DLCO.

In summary, our research aimed to investigate the causal relationship between smoking and DLCO in individuals with IPF via a two-sample MR approach. The research based on summary data of large-scale genome-wide association study (GWAS).

## Methods

### Study design

**Fig. 1:**
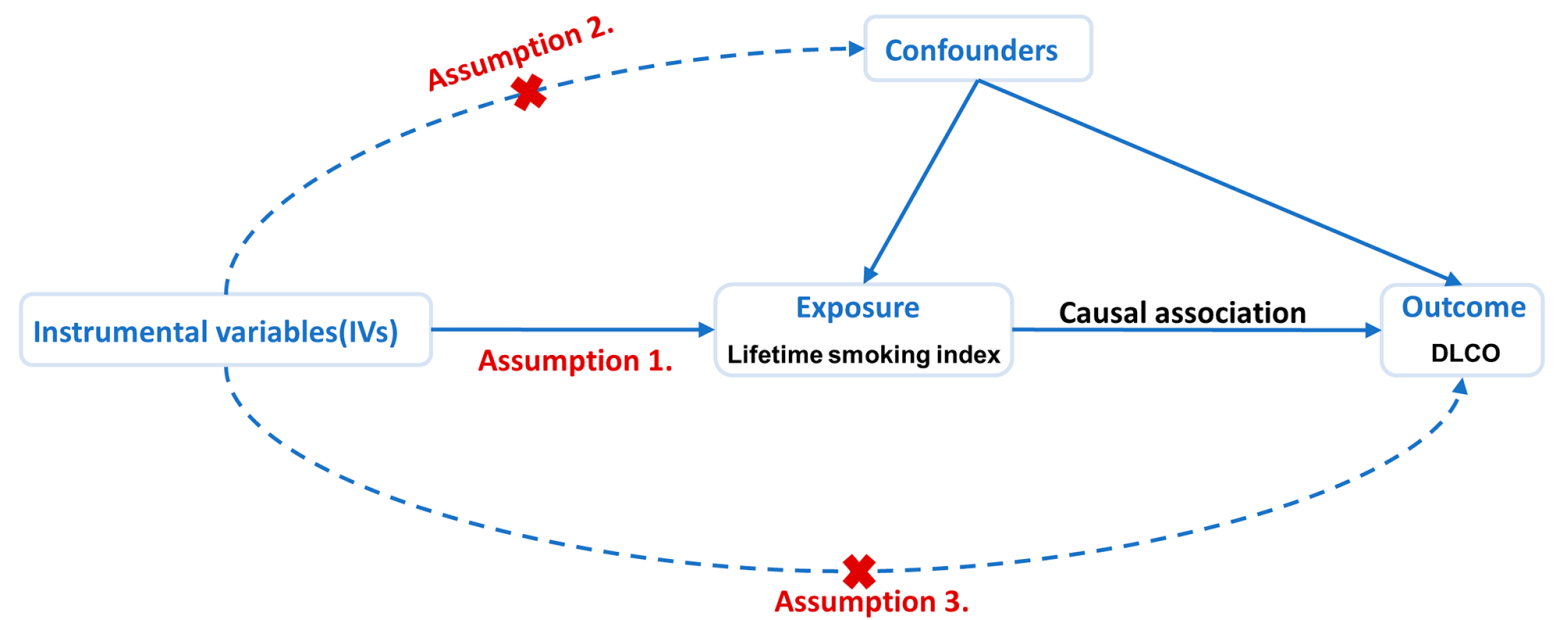
Workflow and the three assumptions for Mendelian randomization analysis

To evaluate the causal link between smoking and DLCO, we executed a two-sample MR analysis. The reliability of instrumental variables (IVs) hinges upon the fulfillment of three fundamental assumptions [12]. First, the genetic variants employed as IVs must be significantly associated with the targeted exposure. Second, these IVs should not be linked to any confounders. Finally, these IVs affect the outcome via alternative pathways (Fig. 1).

### GWAS data sources

The primary metric for quantifying smoking behaviour was the lifetime smoking index (LSI), which was ascertained through a GWAS carried out in the UK Biobank; this study included 462,690 individuals of European descent, as reported by Wootton et al. [13]. The construction of the LSI involved the utilization of self-report questionnaire data regarding smoking intensity, duration, and initiation, following the methodology outlined by Leffondre et al [14]. This approach aimed to provide a more comprehensive representation of smoking habits. The study identified 124 genetic markers associated with the LSI, all of which reached genome-wide significance (*P* < 5 × 10^− 8^) and exhibited minimal linkage disequilibrium (LD) (r2 < 0.001).

The GWAS summary data for DLCO were derived from The Collaborative Group of Genetic Studies of IPF [15]. This comprehensive analysis of 3 cohorts (US, UK, and UUS) included the genotype data of 975 individuals diagnosed with IPF. The patients were diagnosed in accordance with the guidelines established by the American Thoracic Society and the European Respiratory Society.

### Selection of genetic instruments

To ensure the reliability of the IVs used for MR analyses, we adhered to the following criteria. First, we chose single nucleotide polymorphisms (SNPs) associated with smoking-related traits, and the threshold value was *P* < 5 × 10^− 8^. Second, to prevent any LD among all IVs for IPF, we set the clumping parameter to R^2^ < 0.001 and a window size of 10 Mb. Third, during the harmonization process, we removed palindromic SNPs from the IV. Fourth, to mitigate the risk of bias stemming from weak IVs, we calculated the F-statistic $$ (F={beta}^{2}/{se}^{2})$$ [16]. If the F-statistic for IVs greatly exceeded 10, the likelihood of bias from weak IVs was minimal [17]. Moreover, all GWAS data utilized in our MR analyses were limited to individuals of European descent to exclude potential biases from population heterogeneity.

### Mendelian randomization analyses

In our MR analysis, we chose the inverse variance weighted (IVW) method, which combines the Wald ratio for each SNP, as the primary approach, leading to a consolidated causal estimate [18, 19]. To ensure the robustness of our analysis and account for potential pleiotropy, we also conducted sensitivity analyses using several complementary methods. These methods included the weighted mode [20], MR‒Egger [21], weighted median [22], simple mode [23], and MR-Pleiotropy RESidual Sum and Outlier (MR-PRESSO) [24].

### Sensitivity analysis

To identify potential pleiotropy and assess the robustness of our results, we conducted several analyses, including Cochran’s Q statistic [25] and MR‒Egger intercept tests [21]. Specifically, heterogeneity was indicated if the *P* value of the Cochran Q test was less than 0.05. We also assessed horizontal pleiotropy based on the intercept term derived from MR‒Egger regression. In addition, to ascertain whether any single SNP drove the causal estimate, we performed leave-one-out analysis [26].

MR analysis was performed using RStudio (version 4.2.1) with the TwoSampleMR (version 0.5.6) and MRPRESSO (version 1.0) R packages. A significance level of *P* < 0.05 was used to determine statistical significance.

### Ethics

Summary data were used, and ethical approval was not needed.

## Results

### MR estimate

**Fig. 2:**
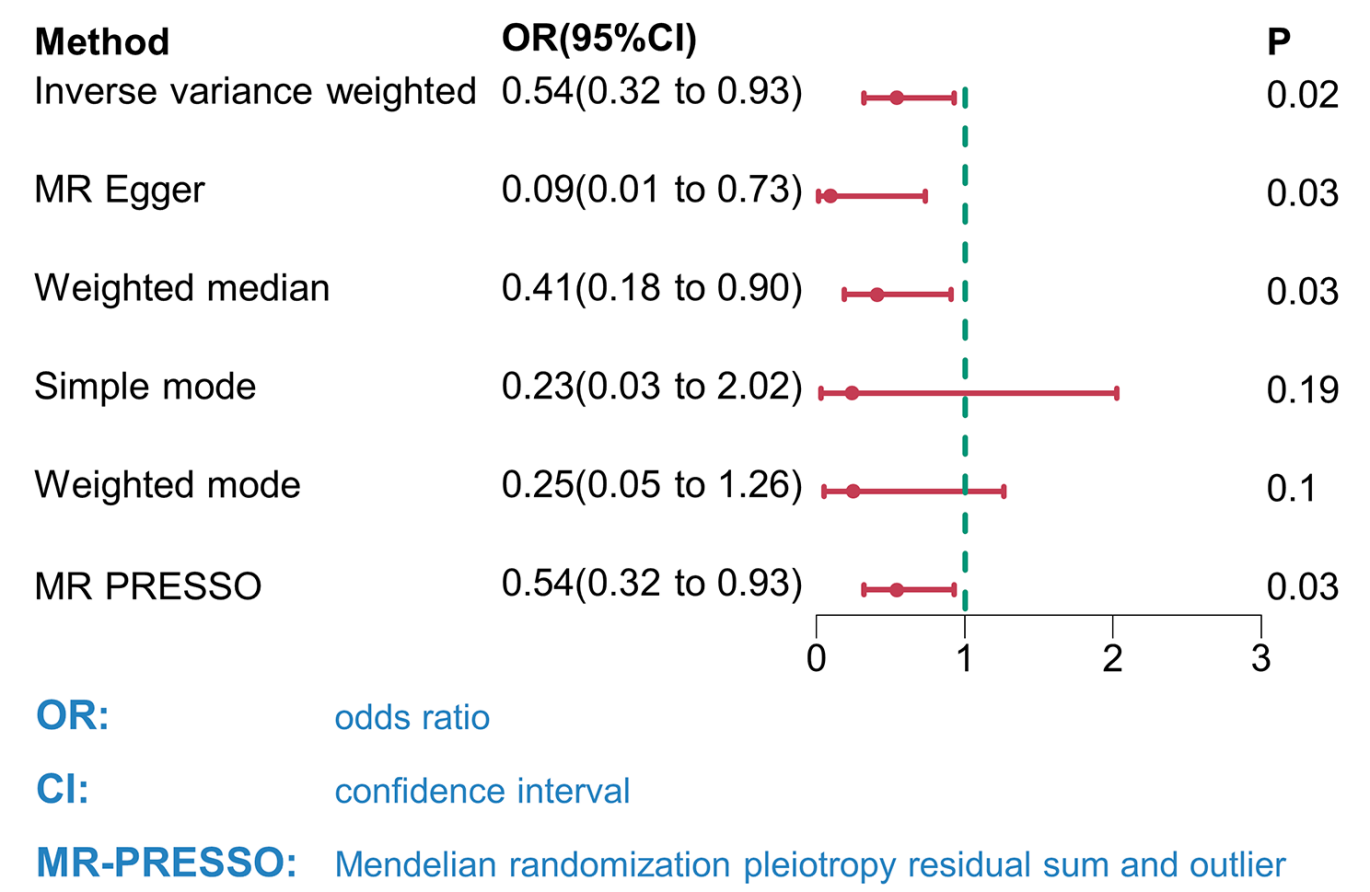
Causal effect of the lifetime smoking index on DLCO

**Fig. 3:**
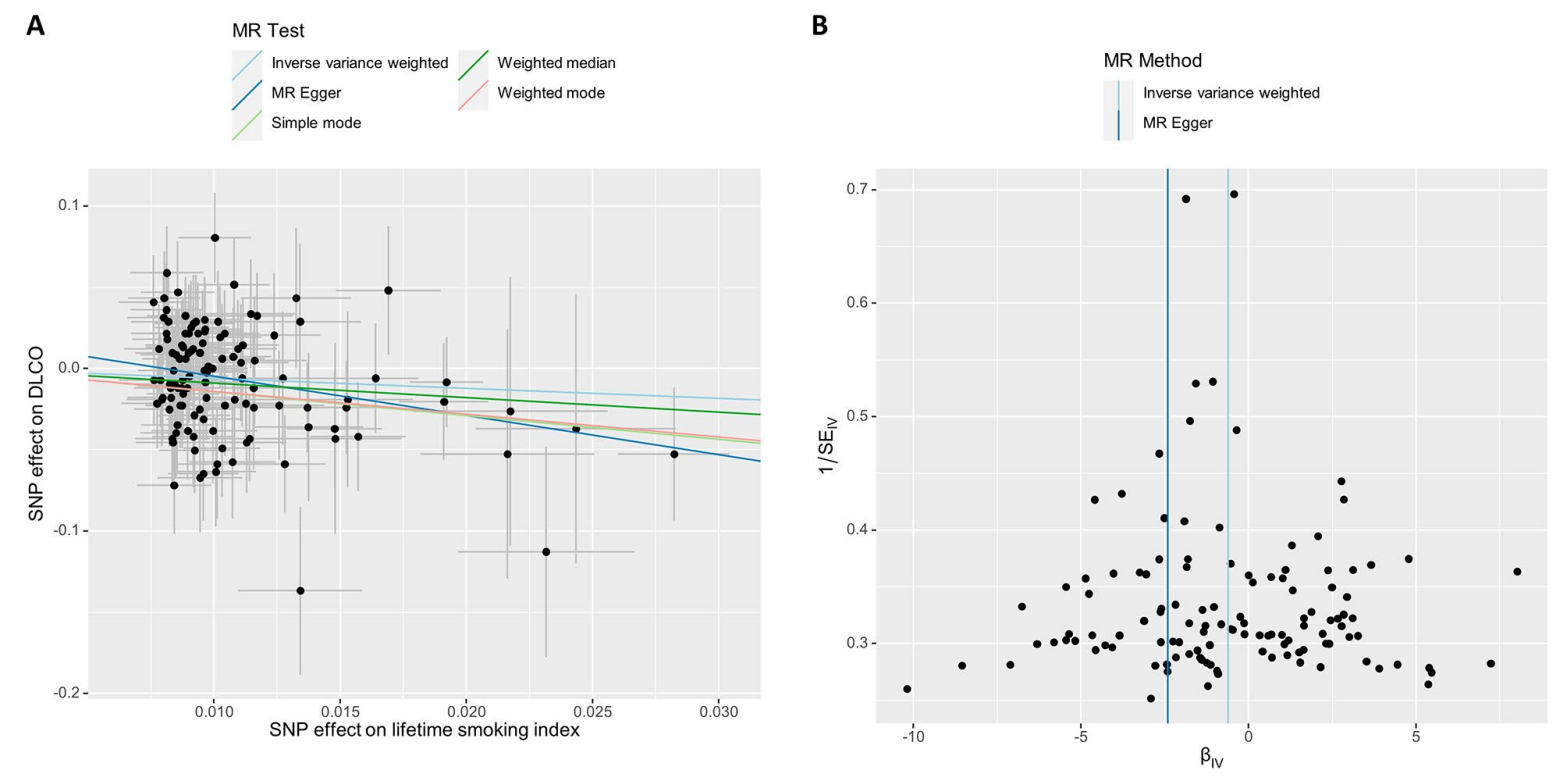
Scatter plot (**A**) of the effect of the lifetime smoking index on DLCO and funnel plot (**B**)

We identified 119 SNPs (Additional file 1) as IVs to investigate the genetic relationship between LSI and DLCO. The F-statistic for each SNP exceeding 30 indicated a low probability of a weak IV. Subsequently, we conducted an MR analysis utilizing these 119 SNPs. The results obtained through the IVW method revealed a causal link between the LSI and DLCO (OR_IVW_ = 0.54, 95% CI 0.32–0.93; *P* = 0.02; Fig. 2). Furthermore, (OR_MR−Egger_ = 0.09, 95% CI 0.01–0.73, *P* = 0.03, Fig. 2; (OR_Weighted median_ = 0.41, 95% CI 0.18–0.90, *P* = 0.03, Fig. 2); (OR_Simple mode_ = 0.23, 95% CI 0.03–2.02, *P* = 0.19, Fig. 2); (OR_Weighted mode_ = 0.25, 95% CI 0.05–1.26, *P* = 0.1, Fig. 2); and (OR_MR−PRESSO_ = 0.54, 95% CI 0.32–0.93, *P* = 0.03, Fig. 2) had consistent directions of effects across all six methods. As illustrated in the scatter plot (Fig. 3A), there was a noticeable decrease in DLCO as the LSI increased.

### Sensitivity analyses

We subsequently conducted sensitivity analyses to assess the robustness of our results. First, Cochran’s Q test demonstrated the absence of heterogeneity among the IVs (P_MR−Egger_ = 0.374, P_IVW_ = 0.325; Table 1). The absence of heterogeneity was also confirmed by the symmetry of the funnel plot (Fig. 3B). Second, there was no indication of overall horizontal pleiotropy across all IVs, as evidenced by the results of both the MR‒Egger regression (*P* = 0.085, Table 1) and the MR-PRESSO global test (*P* = 0.329, Table 1). These results imply that IVs are unlikely to exert their influence on the decrease in DLCO through pathways unrelated to smoking. In the leave-one-out sensitivity analysis, where we systematically excluded one SNP at a time, the results revealed that no specific SNP exerted a significant influence on the DLCO (Additional file 2). As a result, our results remained robust and exhibited no substantial bias.

**Table 1:** Pleiotropy and heterogeneity tests

Tests	Methods	Effect size	*P*
Heterogeneity	Q test (MR Egger)	121.295	0.374
	Q test (IVW)	124.417	0.325
Pleiotropy	Egger regression	0.019	0.085
	MR-PRESSO global test	126.443	0.329

## Discussion

IPF patients have a median survival of 3–5 years after diagnosis but a highly variable clinical course [27]. Lung function in patients with IPF may decline precipitously from the onset of the disease or slowly over the course of the disease, during which acute exacerbations (AEs) occur that can lead to respiratory failure and early death [28]. Therefore, further research into the factors associated with the progression of IPF has become essential, as these factors can enhance the prevention of this condition and decelerate the progression of IPF.

Pulmonary function tests are essential for detecting, diagnosing, and monitoring the progression of IPF. However, given the infancy of computed tomography biomarkers, estimates of disease severity and risk stratification in IPF are still based almost exclusively on functional and physiologic indices, such as forceful lung volume (FVC), diffusing capacity for carbon monoxide (DLCO), and the 6-minute walk test (6MWT), with DLCO considered one of the most valuable parameters for monitoring the progression of IPF. DLCO is considered one of the most valuable pulmonary function test parameters for monitoring the progression of IPF [29–31].. To the best of our knowledge, this is the first study to determine the causal links between smoking and DLCO in patients with IPF based on the MR framework. Our approach drew upon large-scale GWAS data, allowing us to analyse a substantially larger number of cases than did previous observational studies. As expected, our study showed that smoking leads to negative effects on DLCO, which is largely in line with the findings of previous research [7–10]. While the link between smoking and IPF has been established in previous observational studies, our MR analysis offers robust evidence that aligns with the possibility of a causal connection, which is less vulnerable to confounding bias. Nevertheless, because we utilized summary-level data, we were unable to delve into sex-specific associations, indicating the need for future investigations in this area.

The specific mechanisms through which smoking exacerbates a decrease in DLCO have not been identified. Chronic lung inflammation and oxidative stress may be potential pathways mediating the relationship between smoking and reduced DLCO levels. Smoking harms the lungs by inciting chronic inflammation and oxidative stress, thus worsening the progression of IPF [32–35]. It leads to persistent inflammation, disrupts the balance of oxidation, contributes to the buildup of extracellular matrix in the lungs, impairs lung function, hampers gas exchange, and accelerates the deterioration of IPF [32, 36]. Exposure to CS or its extract (CSE) results in the senescence of alveolar epithelial type 2 (AT2) cells, a pivotal process in the progression of lung fibrosis [37]. Several mechanisms drive the CS-induced senescence of AT2 cells, including decreased autophagy, deactivation of the SIRT1 protein, DNA damage, and heightened oxidative stress. In addition, there is growing evidence of a potential correlation between smoking and a variety of IPF prognostic factors (such as MMP-7, SP-A, SP-D, GDF15, and CA-125). For example, higher levels of LOXL2 are associated with poor progression in IPF patients, and there is evidence that LOXL2 is significantly upregulated in patients who smoke. Moreover, SP-D, a serum marker, was found to be higher in smoking patients compared to non-smoking patients [31, 38, 39]. All these imply a potential relationship between CS and multiple IPF prognostic factors. Overall, CS is pivotal in additional damage to the lungs [40]. Concerning its public health implications, our discoveries lend support to the notion that smoking cessation initiatives can serve as an efficacious strategy for mitigating the decrease in DLCO and the ensuing adverse consequences.

Our study offers several notable advantages. First, an inaugural MR investigation was performed to evaluate the causal relationship between elevated smoking and decreased IPF. Second, the robustness of the analysis results was ensured by various sensitivity analysis methods. The study’s limitations must be acknowledged. First, our findings predominantly pertain to participants of European ancestry, and their applicability to populations of different racial backgrounds may be limited. Second, despite the absence of horizontal pleiotropy in our analysis, there may be residual bias due to limited knowledge about the precise functions of most of these SNPs. Third, as our study relied on GWAS summary data instead of individual-level data, it was not possible to stratify our analysis based on other variables, such as age and sex.

## Conclusion

Our study suggested that smoking is an important factor for DLCO decline in IPF patients, which may provide new insights into the progression of IPF. Considering the imperative of delaying disease progression, significant emphasis should be placed on lifestyle management, including smoking cessation as a relevant strategy.

### Electronic supplementary material

Below is the link to the electronic supplementary material.

**Supplementary Material 1: Additional Table.** Instrument variables of lifetime smoking index

**Supplementary Material 2: Additional Figure.** Forest plot (A) and leave-one-out analysis (B) for lifetime smoking index on DLCO

## Data Availability

All the data generated or analysed during this study are included in this published article and its Additional files.

## Abbreviations

AEs: Acute Exacerbations
AT2: Alveolar epithelial type 2
CS: Cigarette smoking
CSE: Cigarette smoking extract
DLCO: Diffusing capacity of the lungs for carbon monoxide
GWAS: Genome-wide association study
ILD: Interstitial Lung Disease
IPF: Idiopathic pulmonary fibrosis
IVs: Instrumental variables
IVW: Inverse variance weighted
LD: Linkage disequilibrium
LSI: Lifetime smoking index
MR-PRESSO: MR pleiotropy residual sum and outlier
MR: Mendelian randomization
SNPs: Single nucleotide polymorphisms

## References

[1] Sgalla G, Biffi A, Richeldi L (2016). Idiopathic pulmonary fibrosis: diagnosis, epidemiology and natural history. Respirology.

[2] Wu JSD, Li Z (2020). Immunity-and-matrix-regulatory cells derived from human embryonic stem cells safely and effectively treat mouse lung injury and fibrosis. Cell Res.

[3] Liu P, Miao K, Zhang L (2020). Curdione ameliorates bleomycin-induced pulmonary fibrosis by repressing TGF-β-induced fibroblast to myofibroblast differentiation. Respir Res.

[4] Gandhi S, Tonelli R, Murray M, Samarelli AV, Spagnolo P. Environmental causes of idiopathic pulmonary fibrosis. *Int J Mol Sci*. 2023;24(22):16481. Published 2023 Nov 18. 10.3390/ijms242216481 10.3390/ijms242216481 PMC10671449 38003670

[5] Kim JS, Ma SF, Ma JZ, et al. Associations of plasma Omega-3 fatty acids with progression and survival in pulmonary fibrosis. Chest Published Online Oct. 2023;20. 10.1016/j.chest.2023.09.035 10.1016/j.chest.2023.09.035 PMC10925547 37866772

[6] Modi P, Cascella M. Diffusing capacity of the lungs for carbon monoxide. In: StatPearls. Treasure Island (FL): StatPearls Publishing; March 13, 2023. 32310609

[7] Schwartz DA, Van Fossen DS, Davis CS (1994). Determinants of progression in idiopathic pulmonary fibrosis. Am J Respir Crit Care Med.

[8] Cherniack RM, Colby TV, Flint A (1995). Correlation of structure and function in idiopathic pulmonary fibrosis. Am J Respir Crit Care Med.

[9] Schwartz DA, Merchant RK, Helmers RA, Gilbert SR, Dayton CS, Hunninghake GW (1991). The influence of cigarette smoking on lung function in patients with idiopathic pulmonary fibrosis. Am Rev Respir Dis.

[10] Kreuter M (2015). IPF, comorbidities and management implications: patient case 1. Sarcoidosis Vasc Diffuse Lung Dis.

[11] Richmond RC, Davey Smith G (2022). Mendelian randomization: concepts and scope. Cold Spring Harb Perspect Med.

[12] Lawlor DA, Harbord RM, Sterne JA, Timpson N, Davey Smith G (2008). Mendelian randomization: using genes as instruments for making causal inferences in epidemiology. Stat Med.

[13] Wootton RE, Richmond RC, Stuijfzand BG (2020). Evidence for causal effects of lifetime smoking on risk for depression and schizophrenia: a Mendelian randomisation study. Psychol Med.

[14] Leffondré K, Abrahamowicz M, Xiao Y, Siemiatycki J (2006). Modelling smoking history using a comprehensive smoking index: application to lung cancer. Stat Med.

[15] Allen RJ, Oldham JM, Jenkins DA (2023). Longitudinal lung function and gas transfer in individuals with idiopathic pulmonary fibrosis: a genome-wide association study. Lancet Respir Med.

[16] Lei W, Yang M, Yuan Z (2023). The causal relationship between physical activity, sedentary time and idiopathic pulmonary fibrosis risk: a Mendelian randomization study. Respir Res.

[17] Burgess S, Thompson SG, CRP CHD Genetics Collaboration (2011). Avoiding bias from weak instruments in Mendelian randomization studies. Int J Epidemiol.

[18] Lin T, Zhou F, Mao H, Xie Z, Jin Y (2023). Vitamin D and idiopathic pulmonary fibrosis: a two-sample Mendelian randomization study. BMC Pulm Med.

[19] Burgess S, Davey Smith G, Davies NM (2023). Guidelines for performing Mendelian randomization investigations: update for summer 2023. Wellcome Open Res.

[20] Hartwig FP, Davey Smith G, Bowden J (2017). Robust inference in summary data Mendelian randomization via the zero modal pleiotropy assumption. Int J Epidemiol.

[21] Bowden J, Davey Smith G, Burgess S (2015). Mendelian randomization with invalid instruments: effect estimation and bias detection through Egger regression. Int J Epidemiol.

[22] Bowden J, Davey Smith G, Haycock PC, Burgess S (2016). Consistent estimation in Mendelian randomization with some Invalid instruments using a weighted median estimator. Genet Epidemiol.

[23] Chen Y, Zhao M, Ji K (2023). Association of nicotine dependence and gut microbiota: a bidirectional two-sample Mendelian randomization study. Front Immunol.

[24] Verbanck M, Chen CY, Neale B, Do R. Detection of widespread horizontal pleiotropy in causal relationships inferred from Mendelian randomization between complex traits and diseases [published correction appears in Nat Genet. 2018;50(8):1196]. Nat Genet. 2018;50(5):693–698. 10.1038/s41588-018-0099-7 10.1038/s41588-018-0099-7 PMC6083837 29686387

[25] Hemani G, Bowden J, Davey Smith G (2018). Evaluating the potential role of pleiotropy in Mendelian randomization studies. Hum Mol Genet.

[26] Burgess S, Bowden J, Fall T, Ingelsson E, Thompson SG (2017). Sensitivity analyses for robust causal inference from Mendelian randomization analyses with multiple genetic variants. Epidemiology.

[27] Harari S, Caminati A (2010). IPF: new insight on pathogenesis and treatment. Allergy.

[28] Martinez FJ, Safrin S, Weycker D (2005). The clinical course of patients with idiopathic pulmonary fibrosis. Ann Intern Med.

[29] Martinez FJ, Flaherty K (2006). Pulmonary function testing in idiopathic interstitial pneumonias. Proc Am Thorac Soc.

[30] Capaccione KM, Wang A, Lee SM (2021). Quantifying normal lung in pulmonary fibrosis: CT analysis and correlation with %DLCO. Clin Imaging.

[31] Karampitsakos T, Juan-Guardela BM, Tzouvelekis A (2023). Precision medicine advances in idiopathic pulmonary fibrosis. EBioMedicine.

[32] Makena P, Kikalova T, Prasad GL, Baxter SA. Oxidative stress and lung fibrosis: towards an adverse outcome pathway. *Int J Mol Sci*. 2023;24(15):12490. Published 2023 Aug 6. 10.3390/ijms241512490 10.3390/ijms241512490 PMC10419527 37569865

[33] Pan M, Zheng Z, Chen Y (2018). Angiotensin-(1–7) attenuated cigarette smoking-related pulmonary fibrosis via improving the impaired autophagy caused by nicotinamide adenine dinucleotide phosphate reduced oxidase 4-dependent reactive oxygen species. Am J Respir Cell Mol Biol.

[34] Liu Y, Lu L, Yang H (2023). Dysregulation of immunity by cigarette smoking promotes inflammation and cancer: a review. Environ Pollut.

[35] Lugg ST, Scott A, Parekh D, Naidu B, Thickett DR (2022). Cigarette smoke exposure and alveolar macrophages: mechanisms for lung disease. Thorax.

[36] Obernolte H, Niehof M, Braubach P (2022). Cigarette smoke alters inflammatory genes and the extracellular matrix - investigations on viable sections of peripheral human lungs. Cell Tissue Res.

[37] Zhang Y, Huang W, Zheng Z (2021). Cigarette smoke-inactivated SIRT1 promotes autophagy-dependent senescence of alveolar epithelial type 2 cells to induce pulmonary fibrosis. Free Radic Biol Med.

[38] Chien JW, Richards TJ, Gibson KF (2014). Serum lysyl oxidase-like 2 levels and idiopathic pulmonary fibrosis disease progression. Eur Respir J.

[39] Maher TM, Oballa E, Simpson JK (2017). An epithelial biomarker signature for idiopathic pulmonary fibrosis: an analysis from the multicentre PROFILE cohort study. Lancet Respir Med.

[40] Douglas D, Keating L, Strykowski R (2023). Tobacco smoking is associated with combined pulmonary fibrosis and emphysema and worse outcomes in interstitial lung disease. Am J Physiol Lung Cell Mol Physiol.

