# Supplementary material for: Genetic association between smoking and DLCO in idiopathic pulmonary fibrosis patients
Source: BMC Pulm Med. 2024 Apr 3;24:163. doi: 10.1186/s12890-024-02974-2 (PMC10993445; doi:10.1186/s12890-024-02974-2)
Supplement: Supplementary file 1 — Supplementary Material 1: Additional Table. Instrument variables of lifetime smoking index [file 12890_2024_2974_MOESM1_ESM.docx]

**Additional Table.** Instrument variables of lifetime smoking index

| **SNP** | **Effect_allele** | **Other_allele** | **Beta** | **EAF** | **SE** | **P** | **F** |
| --- | --- | --- | --- | --- | --- | --- | --- |
| rs10052591 | T | C | 0.0084 | 0.573358 | 0.001402 | 2.10E-09 | 35.88738 |
| rs10226228 | A | G | -0.01141 | 0.62964 | 0.001437 | 2.00E-15 | 63.04317 |
| rs10282292 | C | T | 0.008962 | 0.361838 | 0.001447 | 5.90E-10 | 38.36642 |
| rs1050847 | C | T | 0.00797 | 0.425845 | 0.001405 | 1.40E-08 | 32.18071 |
| rs10823968 | A | T | 0.008135 | 0.632969 | 0.001453 | 2.10E-08 | 31.36765 |
| rs10879871 | T | G | -0.00958 | 0.343436 | 0.001458 | 5.00E-11 | 43.17051 |
| rs10918701 | G | A | 0.008024 | 0.372247 | 0.001432 | 2.10E-08 | 31.40064 |
| rs11210229 | A | G | 0.011702 | 0.383666 | 0.001423 | 2.00E-16 | 67.65115 |
| rs112282219 | G | A | -0.02317 | 0.958949 | 0.003504 | 3.80E-11 | 43.70557 |
| rs11255908 | T | G | -0.01007 | 0.743485 | 0.001588 | 2.30E-10 | 40.21803 |
| rs113382419 | C | A | -0.02823 | 0.889325 | 0.002214 | 3.00E-37 | 162.66 |
| rs11783093 | C | T | 0.01571 | 0.838811 | 0.001897 | 1.20E-16 | 68.61574 |
| rs11861214 | G | T | 0.009451 | 0.78364 | 0.001685 | 2.00E-08 | 31.47519 |
| rs11948770 | T | C | -0.01024 | 0.768248 | 0.001645 | 4.90E-10 | 38.71114 |
| rs12202536 | A | G | -0.00823 | 0.512754 | 0.001386 | 2.80E-09 | 35.29226 |
| rs1221148 | C | G | 0.009165 | 0.587062 | 0.001407 | 7.30E-11 | 42.4241 |
| rs1246265 | T | C | -0.00887 | 0.30456 | 0.001509 | 4.20E-09 | 34.51153 |
| rs12481282 | G | C | -0.00894 | 0.722343 | 0.001549 | 7.80E-09 | 33.32129 |
| rs12623702 | A | G | -0.00977 | 0.613375 | 0.001428 | 7.70E-12 | 46.8407 |
| rs12708665 | A | G | -0.00909 | 0.284632 | 0.001539 | 3.50E-09 | 34.87004 |
| rs12831617 | C | T | -0.00918 | 0.764305 | 0.001633 | 1.90E-08 | 31.62807 |
| rs12967855 | A | G | 0.008189 | 0.331225 | 0.001479 | 3.10E-08 | 30.64036 |
| rs13009008 | A | G | 0.008633 | 0.327713 | 0.001473 | 4.60E-09 | 34.33356 |
| rs13016665 | C | A | -0.00849 | 0.576725 | 0.001412 | 1.80E-09 | 36.14166 |
| rs13153393 | A | G | -0.01375 | 0.883937 | 0.002173 | 2.50E-10 | 40.00004 |
| rs13296519 | G | T | -0.0097 | 0.606385 | 0.001419 | 8.10E-12 | 46.7484 |
| rs136233 | A | G | -0.00996 | 0.809084 | 0.001769 | 1.80E-08 | 31.66926 |
| rs147412694 | G | A | -0.01157 | 0.850009 | 0.001949 | 2.90E-09 | 35.26909 |
| rs17309874 | G | A | -0.01129 | 0.740357 | 0.001582 | 9.70E-13 | 50.90736 |
| rs17553262 | A | C | -0.01273 | 0.884605 | 0.002181 | 5.30E-09 | 34.06522 |
| rs17576594 | G | A | 0.01095 | 0.7235 | 0.001552 | 1.70E-12 | 49.80697 |
| rs1922018 | C | T | 0.010033 | 0.364422 | 0.001438 | 3.00E-12 | 48.69695 |
| rs1931263 | G | T | -0.00761 | 0.510321 | 0.001386 | 4.00E-08 | 30.14563 |
| rs1933270 | T | G | 0.009218 | 0.363726 | 0.001438 | 1.50E-10 | 41.09004 |
| rs202645 | A | G | -0.01016 | 0.202939 | 0.001725 | 3.90E-09 | 34.69023 |
| rs2062882 | G | A | -0.00811 | 0.586782 | 0.00142 | 1.10E-08 | 32.62191 |
| rs2254710 | C | A | 0.008996 | 0.236356 | 0.001631 | 3.50E-08 | 30.43026 |
| rs245774 | A | G | -0.00902 | 0.271691 | 0.00156 | 7.40E-09 | 33.41454 |
| rs2675638 | G | A | 0.008499 | 0.58057 | 0.0014 | 1.30E-09 | 36.8409 |
| rs2838834 | C | T | -0.00936 | 0.699459 | 0.001515 | 6.30E-10 | 38.21632 |
| rs28485305 | C | T | 0.008007 | 0.631482 | 0.001439 | 2.60E-08 | 30.96419 |
| rs28635466 | G | A | 0.008442 | 0.696005 | 0.001505 | 2.00E-08 | 31.47475 |
| rs2867112 | T | G | 0.014783 | 0.834596 | 0.001887 | 4.80E-15 | 61.34466 |
| rs2890772 | G | T | -0.0137 | 0.413181 | 0.001407 | 2.10E-22 | 94.81841 |
| rs2894808 | T | A | -0.0153 | 0.922121 | 0.002591 | 3.50E-09 | 34.85656 |
| rs317021 | T | A | -0.01157 | 0.814287 | 0.001791 | 1.10E-10 | 41.71703 |
| rs326341 | G | A | 0.009435 | 0.524943 | 0.001392 | 1.20E-11 | 45.91265 |
| rs329120 | C | T | 0.009657 | 0.580672 | 0.001405 | 6.30E-12 | 47.23672 |
| rs34866095 | A | G | -0.00857 | 0.686187 | 0.001506 | 1.20E-08 | 32.41564 |
| rs348809 | A | G | -0.00828 | 0.347637 | 0.001456 | 1.30E-08 | 32.36872 |
| rs35169606 | T | G | 0.008776 | 0.612204 | 0.001444 | 1.20E-09 | 36.94671 |
| rs35175834 | G | A | -0.0164 | 0.78844 | 0.001698 | 4.60E-22 | 93.26988 |
| rs35343344 | C | A | 0.009181 | 0.732713 | 0.001596 | 8.80E-09 | 33.09699 |
| rs359243 | T | C | -0.00872 | 0.392886 | 0.001425 | 9.50E-10 | 37.42318 |
| rs369230 | G | T | -0.00909 | 0.307565 | 0.001511 | 1.80E-09 | 36.18146 |
| rs3742365 | T | C | -0.01079 | 0.595136 | 0.001416 | 2.50E-14 | 58.10468 |
| rs3811038 | T | C | -0.00954 | 0.723852 | 0.001557 | 8.90E-10 | 37.55716 |
| rs3896224 | A | G | 0.009627 | 0.585286 | 0.001418 | 1.10E-11 | 46.09724 |
| rs421983 | T | C | 0.008707 | 0.519028 | 0.001386 | 3.30E-10 | 39.49098 |
| rs4391802 | A | G | 0.010319 | 0.707464 | 0.001528 | 1.40E-11 | 45.61414 |
| rs4473348 | A | T | -0.01043 | 0.249978 | 0.001597 | 6.40E-11 | 42.68117 |
| rs4543592 | T | C | -0.00866 | 0.519912 | 0.001389 | 4.50E-10 | 38.88772 |
| rs4568549 | C | A | -0.00762 | 0.503149 | 0.001387 | 3.90E-08 | 30.1928 |
| rs4571506 | C | T | 0.007877 | 0.539523 | 0.001392 | 1.50E-08 | 32.03458 |
| rs4671357 | T | C | -0.00944 | 0.518819 | 0.00139 | 1.10E-11 | 46.14574 |
| rs4731925 | C | T | -0.00829 | 0.315607 | 0.00149 | 2.60E-08 | 30.96411 |
| rs4814873 | C | T | 0.009712 | 0.76659 | 0.001636 | 2.90E-09 | 35.22629 |
| rs4949465 | T | C | -0.01161 | 0.869552 | 0.002058 | 1.70E-08 | 31.80443 |
| rs4957528 | A | C | -0.01012 | 0.208497 | 0.001722 | 4.20E-09 | 34.55006 |
| rs530916 | A | G | -0.00782 | 0.439502 | 0.001395 | 2.00E-08 | 31.46444 |
| rs549845 | G | A | 0.011261 | 0.301161 | 0.001509 | 8.30E-14 | 55.72449 |
| rs57611503 | G | A | 0.007743 | 0.484578 | 0.00141 | 4.00E-08 | 30.14876 |
| rs6011779 | C | T | 0.019115 | 0.191239 | 0.001764 | 2.30E-27 | 117.4729 |
| rs60952428 | T | C | 0.013411 | 0.909149 | 0.002419 | 3.00E-08 | 30.72739 |
| rs6119897 | G | A | -0.0128 | 0.761716 | 0.001627 | 3.60E-15 | 61.92718 |
| rs61796681 | A | T | -0.01342 | 0.912365 | 0.002448 | 4.20E-08 | 30.04782 |
| rs62098013 | G | A | -0.00857 | 0.63991 | 0.001457 | 4.10E-09 | 34.58151 |
| rs62135536 | C | T | 0.024344 | 0.968256 | 0.003962 | 8.00E-10 | 37.76121 |
| rs62155874 | A | G | -0.01691 | 0.87336 | 0.002085 | 5.20E-16 | 65.72715 |
| rs62175972 | T | C | 0.021746 | 0.965939 | 0.003857 | 1.70E-08 | 31.79165 |
| rs624833 | T | G | 0.009286 | 0.694697 | 0.001504 | 6.60E-10 | 38.13784 |
| rs6562474 | C | G | 0.008369 | 0.650606 | 0.001461 | 1.00E-08 | 32.80966 |
| rs6598539 | T | C | -0.00815 | 0.488672 | 0.001389 | 4.50E-09 | 34.41233 |
| rs6741228 | T | C | 0.007933 | 0.433309 | 0.001404 | 1.60E-08 | 31.9411 |
| rs67596067 | G | A | -0.00888 | 0.648812 | 0.001458 | 1.20E-09 | 37.04486 |
| rs6778080 | T | C | 0.011114 | 0.267372 | 0.001566 | 1.30E-12 | 50.3557 |
| rs6779302 | G | T | -0.00875 | 0.632921 | 0.001439 | 1.20E-09 | 36.98494 |
| rs6935954 | A | G | 0.009582 | 0.421108 | 0.001402 | 8.20E-12 | 46.7131 |
| rs6962772 | A | G | 0.011064 | 0.845551 | 0.001916 | 7.80E-09 | 33.32954 |
| rs7039819 | G | A | 0.008734 | 0.427307 | 0.001405 | 5.10E-10 | 38.64563 |
| rs7077678 | C | T | 0.00855 | 0.623429 | 0.001436 | 2.60E-09 | 35.465 |
| rs71367545 | G | A | -0.01032 | 0.790533 | 0.001704 | 1.40E-09 | 36.68571 |
| rs7155595 | A | C | -0.00885 | 0.674282 | 0.001485 | 2.50E-09 | 35.52596 |
| rs71627581 | G | A | 0.013257 | 0.888811 | 0.002199 | 1.60E-09 | 36.35201 |
| rs72674867 | A | T | 0.008988 | 0.764568 | 0.001634 | 3.80E-08 | 30.25592 |
| rs72678864 | G | A | 0.012383 | 0.828591 | 0.001839 | 1.60E-11 | 45.35477 |
| rs7297175 | T | C | -0.00812 | 0.431355 | 0.001399 | 6.60E-09 | 33.64937 |
| rs732083 | G | A | 0.008348 | 0.333362 | 0.001473 | 1.50E-08 | 32.11578 |
| rs73220544 | A | C | -0.01082 | 0.842295 | 0.001913 | 1.50E-08 | 31.99764 |
| rs7333559 | G | A | 0.01074 | 0.211711 | 0.001707 | 3.20E-10 | 39.57857 |
| rs74086911 | G | A | 0.014802 | 0.925459 | 0.002642 | 2.10E-08 | 31.38997 |
| rs7519626 | C | T | 0.00842 | 0.323676 | 0.001479 | 1.20E-08 | 32.41488 |
| rs7528604 | G | A | 0.009653 | 0.565613 | 0.001401 | 5.70E-12 | 47.44236 |
| rs7553348 | G | A | 0.009634 | 0.437656 | 0.001396 | 5.20E-12 | 47.59639 |
| rs7569203 | A | C | -0.01076 | 0.688725 | 0.0015 | 7.40E-13 | 51.43821 |
| rs75742406 | G | A | 0.009619 | 0.738854 | 0.001585 | 1.30E-09 | 36.84015 |
| rs76608582 | C | A | 0.021632 | 0.953011 | 0.00344 | 3.20E-10 | 39.54184 |
| rs7766610 | C | A | 0.012584 | 0.182634 | 0.001793 | 2.20E-12 | 49.26657 |
| rs7807019 | A | G | -0.01042 | 0.540327 | 0.001391 | 6.70E-14 | 56.15781 |
| rs8042134 | T | G | -0.00994 | 0.541083 | 0.001401 | 1.30E-12 | 50.34465 |
| rs8042849 | C | T | 0.019216 | 0.34229 | 0.001462 | 1.80E-39 | 172.8026 |
| rs812887 | A | G | 0.008248 | 0.414385 | 0.00141 | 5.00E-09 | 34.20679 |
| rs860326 | C | T | 0.008338 | 0.427696 | 0.001402 | 2.70E-09 | 35.35495 |
| rs8614 | C | A | -0.01146 | 0.817489 | 0.001797 | 1.80E-10 | 40.68792 |
| rs889398 | C | T | 0.009241 | 0.588005 | 0.001414 | 6.30E-11 | 42.72811 |
| rs9435340 | T | A | 0.008348 | 0.344234 | 0.001464 | 1.20E-08 | 32.50216 |
| rs9842947 | C | T | -0.00877 | 0.326165 | 0.001481 | 3.10E-09 | 35.09414 |
| rs986391 | G | A | 0.011139 | 0.366555 | 0.001438 | 9.40E-15 | 60.00942 |
| rs9919670 | G | A | -0.01524 | 0.612185 | 0.001421 | 7.60E-27 | 115.0569 |
