# Supplementary material for: Genetic association between smoking and DLCO in idiopathic pulmonary fibrosis patients
Source: BMC Pulm Med. 2024 Apr 3;24:163. doi: 10.1186/s12890-024-02974-2 (PMC10993445; doi:10.1186/s12890-024-02974-2)
Supplement: Supplementary file 2 — Supplementary Material 2: Additional Figure. Forest plot (A) and leave-one-out analysis (B) for lifetime smoking index on DLCO [file 12890_2024_2974_MOESM2_ESM.pdf]

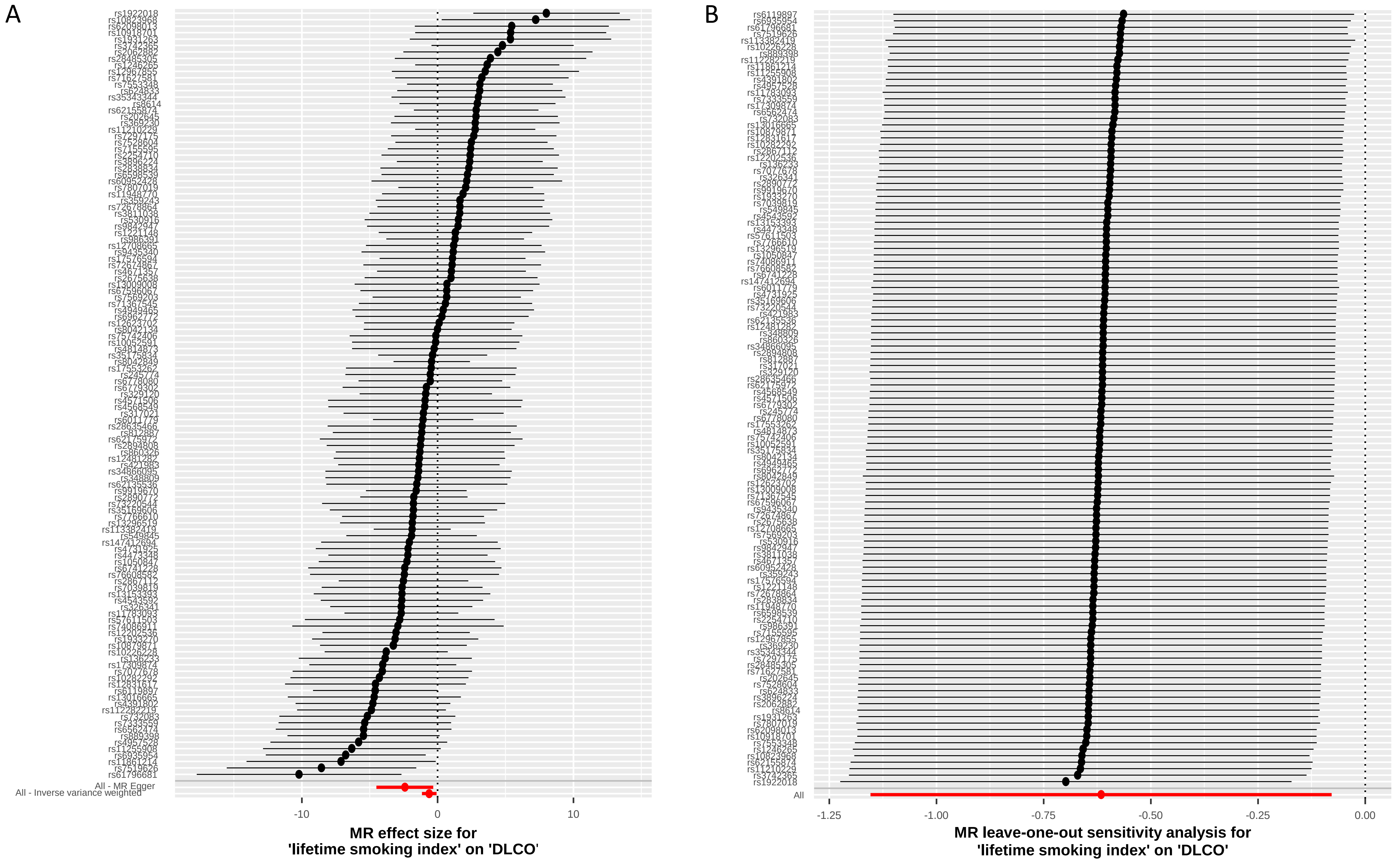

Additional Figure. Forest plot (A) and leave-one-out analysis (B) for lifetime smoking index on DLCO
